# Thinner Corneas Appear to Have More Striking Effects of Corneal Collagen Crosslinking in Patients with Progressive Keratoconus

**DOI:** 10.1155/2017/6490915

**Published:** 2017-03-14

**Authors:** Yunfei Han, Yanyun Xu, Wei Zhu, Yuling Liu, Zhen Liu, Xiaoxiao Dou, Guoying Mu

**Affiliations:** ^1^Department of Ophthalmology, Shandong Provincial Hospital Affiliated to Shandong University, Jinan 250021, China; ^2^Department of Ophthalmology, The Second People's Hospital of Liaocheng, Linqing 252600, China; ^3^Department of Ophthalmology, Jinan Central Hospital Affiliated to Shandong University, Jinan 250013, China

## Abstract

*Purpose.* To analyze the outcomes and difference after UVA/riboflavin corneal collagen crosslinking (CXL) in four different corneal thickness groups of patients with progressive keratoconus. *Methods.* Retrospective study. Eyes with progressive keratoconus after CXL were divided into 4 subgroups as follows: group 1, thinnest corneal thickness (TCT) ≤ 400 *µ*m; group 2, 400 *µ*m < TCT ≤ 450 *µ*m; group 3, 450 *µ*m < TCT ≤ 500 *µ*m; group 4, TCT ≥ 500 *µ*m. Baseline, 6-month, and 12-month visual acuity, corneal topography, TCT, and endothelial cell density were evaluated. *Results.* The analysis included 123 eyes of 101 patients. At 6 and 12 months after CXL, there was a mean improvement about visual acuity and keratometry values in all patients. There was a reduction in the change of maximum keratometry (*K*_max_) with the increase of TCT. After 1 year of treatment, it was 3.04 ± 0.75 D in group 1, 2.38 ± 0.51 D in group 2, 1.57 ± 0.35 D in group 3, and 0.31 ± 0.20 D in group 4. *Conclusion.* CXL is successful in halting the progression of keratoconus and there was a negative linear correlation between TCT and *K*_max_. Advanced cases of progressive keratoconus seemed to obtain more benefits from the flatting effects of CXL.

## 1. Introduction

Keratoconus (KC) is a corneal noninflammatory degeneration characterized by bilateral conical protrusion and corneal thinning. It can vary from slightly irregular astigmatism to severe visual impairment resulting from the increased corneal protrusion and subepithelial scarring [1]. KC leads to biomechanical alterations, but its underlying pathogenesis is not clear. Corneal collagen crosslinking (CXL) is a noninvasive therapeutic approach to improve the biomechanical and biochemical properties of the cornea and is the only treatment to date that addresses the pathophysiology of KC [2]. In the traditional view, CXL is generally performed on the eyes with a corneal thickness of at least 400 *μ*m to avoid the cytotoxic effects of the UVA on the corneal endothelium, lens, and other intraocular tissues. In fact, in many patients with advanced stages of KC, corneal thickness usually exceeds that of the standard. Hence, various measures have been suggested to circumvent, for example, using hypoosmolar riboflavin solution, customized epithelial debridement technique, and contact lens-assisted CXL [3]. In terms of safety and effectiveness, these techniques also achieved good results. We were interested in the potential correlation after CXL in different corneal thickness groups and whether there was an association between pre-CXL corneal thickness and the change of *K*_max_ (Δ*K*_max_ defined as the difference in *K*_max_ between the preoperative and the last registered visit). So, we did this analysis.

## 2. Patients and Methods

### 2.1. Patients

A retrospective cohort study was performed at the Department of Ophthalmology, Shandong Provincial Hospital affiliated to Shandong University. All patients were diagnosed as progressive keratoconus. 101 patients (123 eyes) were enrolled in this analysis from March 2014 to September 2015. The patients were divided into 4 subgroups as follows: group 1, keratoconus with thinnest corneal thickness (TCT) less than 400 *μ*m; group 2, keratoconus with TCT between 400 *μ*m and 450 *μ*m; group 3, keratoconus with TCT between 450 *μ*m and 500 *μ*m, and group 4, keratoconus with TCT 500 *μ*m or more. The progressive keratoconus was defined as 1 or more of the following changes over a period of 24 months: an increase of 1.00 diopter (D) or more in the steepest keratometry (K) measurement, an increase of 1.00 D or more in manifest cylinder, and an increase of 0.50 D or more in manifest refraction spherical equivalent (MRSE) [4].

Exclusion criteria included a history of corneal surgery, corneal pachymetry less than 340 *μ*m, a history of chemical injury or delayed epithelial healing, and pregnancy or lactation during the course of the study.

### 2.2. Surgical Technique

The procedure was performed under sterile conditions in the outpatient operation room. After topical anesthesia with proparacaine hydrochloride 0.5% eye drops (Alcaine, Alcon Laboratories Inc), corneal epithelium was mechanically removed within the central 9 mm diameter area using a mechanical microkeratome (Amadeus II, Ziemers, Switzerland). 0.1% solution of riboflavin (Ricrolin; Sooft Italia S.p.A, Montegiorgio, Italy) was applied to the denuded stroma every 3 minutes for 30 minutes. Saturation of the corneal stroma and the presence of riboflavin in the anterior chamber were monitored using the blue light of the slit lamp. In eyes with a denuded corneal thickness below 400 *μ*m measured with optical coherence tomography (OCT, Cirrus HD-OCT 4000; Carl Zeiss Meditec Inc, Hacienda Drive, Dublin, USA), 0.1% hypoosmolar riboflavin solution, which was generated by diluting vitamin B2-riboflavin-5-phosphate 0.5% (Shandong Fangming Pharmaceutical Limited by Share Ltd., Shandong, China) with physiological salt solution (sodium chloride 0.9% solution; 310 mOsmol/L; Sichuan Kelun Pharmaceutical Limited by Share Ltd., Sichuan, China), was administered every 10 seconds for 2 minutes until 400 *μ*m was achieved. Subsequently, the cornea was irradiated with a calibrated ultraviolet A light source (370 nm, 3.0 mW/cm^2^, UV-X illumination system version 1000, UVXTM, IROCAG, Zurich, Switzerland) for 30 minutes at a distance of 5 cm. During ultraviolet A irradiation, isotonic or hypoosmolar riboflavin was applied every 3 minutes to maintain the riboflavin saturation in the corneal stroma. Postoperative treatment comprised therapeutic bandage contact lens, antibiotic eye drops 4 times daily for 1 week, and fluorometholone eye drops 0.1% 4 times a day for 4 weeks.

### 2.3. Follow-Up Evaluation

Patients were examined at baseline, 6, and 12 months after CXL. All patients were requested to discontinue contact lens wear before each evaluation. Examinations included UCVA, BCVA, *K*_max_, *K*1, *K*2, slit lamp evaluation, TCT (corneal topography, Allegro Topolyzer Vario, WaveLight GmbH, Erlangen, Germany), IOP (Non-contact Tonometer, Canon Full Auto Tonometer TX-F, Japan), and endothelial cell density (ECD, Specular Microscope SP-3000P, Japan).

### 2.4. Statistical Analysis

Statistical analysis was performed using SPSS software version 20.0 (SPSS, Inc.). Continuous variables were expressed with means ± standard deviation or median according to their normal distribution or not. Comparison between four groups was performed using one-factor ANOVA or Kruskal-Wallis *H* test. Comparison before and after operation was performed using paired *t*-test or Wilcoxon signed-rank test. Correlation between preoperative corneal thickness and Δ*K*_max_ was tested by the Spearman rank correlation. A *P* value less than 0.05 was considered statistically significant.

## 3. Results

### 3.1. Patient Demographics

123 eyes of 101 patients were treated, with a male to female preponderance of 3 : 1; mean age was 22.7 ± 5.1 years (range, 14–37 years); mean TCT was 445.5 *μ*m (range, 348–544 *μ*m). There were 31 patients (25.2%) in group 1, 41 patients (33.3%) in group 2, 36 patients (29.3%) in group 3, 15 patients (12.2%) in group 4.. Post-therapy follow-up duration was 12 months.

### 3.2. Six-Month Outcomes of CXL

Table 1 shows the outcomes of surgery at 6-month follow-up in the overall group. Before treatment, the mean TCT was 445.5 *μ*m (SD ± 48.7), mean *K*_max_ was 58.99 D (SD ± 9.99), mean *K*1 was 47.14 D (SD ± 4.45), mean *K*2 was 50.33 D (SD ± 5.73), UCVA was 0.80 logMAR (SD ± 0.36), and the BCVA was 0.35 logMAR (SD ± 0.33). Six months after treatment, *K*_max_ was significantly decreased to a mean value of 57.24 D; *K*1 and *K*2 decreased to 46.83 D and 49.84 D, respectively. There was a tendency toward visual acuity improvement, reaching 0.72 logMAR (SD ± 0.36) for UCVA and 0.25 logMAR (SD ± 0.28) for BCVA. However, statistical analysis of IOP and ECD also showed statistically significant variation before and after treatment during the follow-up period (*P* = 0.016, *P* < 0.001, resp.). The initial mean TCT was 445.7 *μ*m (SD ± 48.65). Six months after CXL, there was a significant reduction with a value of 421.6 *μ*m (SD ± 48.2, *P* < 0.001).

### 3.3. One-Year Outcomes of CXL

Table 1 shows the outcomes of surgery at 12-month follow-up in the overall group. One year after treatment, *K*_max_ was significantly decreased to a mean value of 57.05 D; *K*1 and *K*2 decreased to 46.56 D and 49.56 D, respectively. There was a tendency toward visual acuity improvement, reaching 0.68 logMAR (SD ± 0.35) for UCVA and 0.20 logMAR (SD ± 0.26) for BCVA. However, statistical analysis of IOP and ECD also showed statistically significant variation before and after treatment during the follow-up period (*P* < 0.001 and *P* = 0.001). The initial mean TCT was 445.5 *μ*m (SD ± 48.7). Twelve months after treatment, there was a significant reduction with a value of 422.3 *μ*m (SD ± 45.9, *P* < 0.001).

Table 2 shows the changes of parameters compared with baseline by groups. There was a mean improvement about UCVA, BCVA, *K*1, *K*2, and *K*_max_ in the four groups. In the meanwhile, there was a decrease in TCT and ECD.

### 3.4. Comparison of Δ*K*_max_ between the Four Study Groups

**Figure 1:**
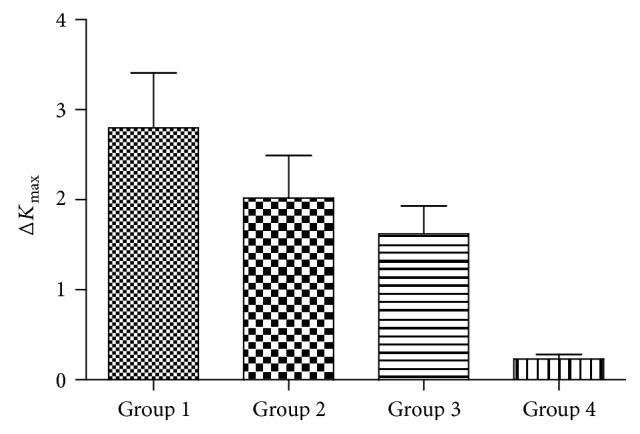
Comparison of Δ*K*_max_ between four groups after six months of treatment.

**Figure 2:**
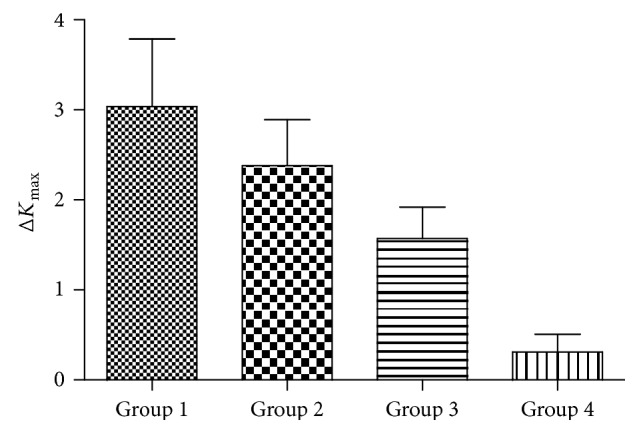
Comparison of Δ*K*_max_ between four groups after 1 year of treatment.

In the subgroup of eyes with keratoconus, analysis showed a trend toward a reduction in Δ*K*_max_ with the increase of TCT. After six months of CXL, the Δ*K*_max_ was 2.80 D (SD ± 0.61) in group 1, 2.02 D (SD ± 0.47) in group 2, 1.62 D (SD ± 0.31) in group 3, and 0.05 D (SD ± 0.23) in group 4. The difference between the four groups was statistically significant. One year after CXL, the Δ*K*_max_ was 3.04 D (SD ± 0.75) in group 1, 2.38 D (SD ± 0.51) in group 2, 1.57 D (SD ± 0.35) in group 3, and 0.31 D (SD ± 0.20) in group 4. The difference between the four groups was statistically significant (Tables 3 and 4, Figures 1 and 2).

### 3.5. The Relationship between Pre-CXL TCT and the Δ*K*_max_

**Figure 3:**
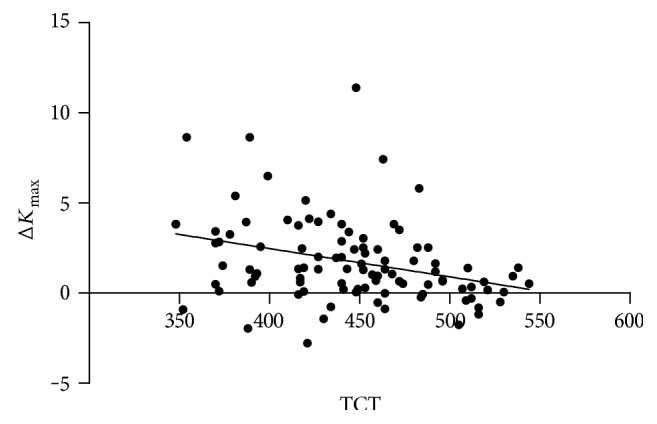
The scatter plot between TCT and Δ*K*_max_ after 6 months of treatment.

**Figure 4:**
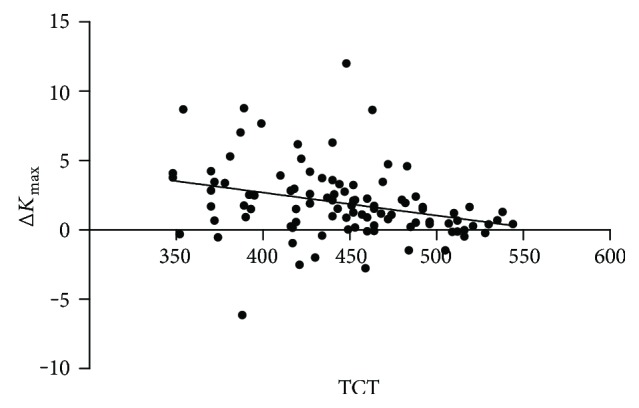
The scatter plot between TCT and Δ*K*_max_ after 12 months of treatment.

In order to study the relationship between the two variables, we drew the scatter plot and then fit a line with linear regression and found that they had a negative linear correlation (Figure 3 and Figure 4). Also, this correlation was statistically significant (correlation coefficient was −0.326 and −0.383, resp., *P* < 0.001).

In order to study the change of Δ*K*_max_ along with TCT, we used regression analysis to access. Set TCT as the independent variable *x*, Δ*K*_max_ as the dependent variable *y*. The regression equation was as follows: *y* = 9.311 − 0.017 × *x*. That is, in the TCT of each increase in one unit (*μ*m), the average Δ*K*_max_ will decline 0.017 D.

## 4. Discussion

In 2003, Wollensak et al. first reported the corneal collagen crosslinking induced by riboflavin/ultraviolet A for the treatment of keratoconus [5]. Since then, a lot of similar studies began to emerge. Several reports have established the CXL as being safe and effective. For example, after 36 months follow-up, Wittig-Silva et al. reported 94 patients (100 eyes) who had CXL gained a significant flattening compared with controls [6]. In France, 42 eyes (68.8%) coming from 142 patients (142 eyes) diagnosed with keratoconus had stopped progression after CXL treatment. Viswanathan and Males found *K*_max_ of 51 eyes decreased about 0.96 D [7]. Similarly, our study also demonstrated the efficacy of CXL with UVA-riboflavin in the stabilization of keratoconus and recovery of visual acuity. Before treatment, the mean *K*_max_ was 58.99 D (SD ± 9.99), mean *K*1 was 47.14 D (SD ± 4.45), mean *K*2 was 50.33 D (SD ± 5.73), UCVA was 0.80 logMAR (SD ± 0.36), and the BCVA was 0.35 logMAR (SD ± 0.33). One year after treatment, *K*_max_ was significantly decreased to a mean value of 57.05 D, *K*1 and *K*2 decreased to 46.56 D and 49.56 D, respectively, and UCVA increased to 1.68 logMAR (SD ± 0.35) and BCVA to 0.20 logMAR (SD ± 0.26).

It has been reported that CXL increases stiffness by a factor of 1.5 [8]. Research studies indicated the cytotoxic UVA irradiance level for keratocytes as 0.5 mW/cm^2^ and demonstrated a variable degree of apoptosis and cell loss in the anterior 250–300 *μ*m of the corneal stroma with a surface irradiance of 3 mW/cm^2^ [9]. An irradiance level of 0.37 mW/cm^2^ at the endothelial cell layer is cytotoxic for the endothelial cells [10, 11]. When the surface irradiance is 3 mW/cm^2^, the 0.37 mW/cm^2^ irradiance reached a depth of 300 *μ*m in the anterior of the cornea. So we determined the lower limit of the thinnest pachymetry as 340 *μ*m with epithelium taking into account.

As is known to all, the aim of CXL is to halt progression of keratoconus and to preserve usable visual acuity. So, the maximum *K* (*K*_max_) value derived from corneal topography analysis is very important and often been used to evaluate the efficacy of treatment. Flattening of *K*_max_ is a measure for treatment success, and steepening is an indication of poorer procedure efficacy. This is the focus of our analysis and the place of interest. Most of the studies reported a *K*_max_ reduction of 1-2 D after 1-year post-CXL. Hashemi et al. reported that *K*_max_ and *K*_mean_ decreased slightly (by 0.16 D and 0.1 D, resp.) at 5 years after the CXL procedure [12]. Hersh et al. reported a reduction of 1.70 D based on their RCT of 48 eyes [4]. Henriquez et al. reported a 2.66 D reduction in *K*_max_ based on their randomized prospective comparative study of 10 eyes [13].

In our retrospective analysis, these cases were divided into four groups according to TCT. After six months of treatment, it was 2.80 D (SD ± 0.61) in group 1, 2.02 D (SD ± 0.47) in group 2, 1.62 D (SD ± 0.31) in group 3, and 0.05 D (SD ± 0.23) in group 4. After 1 year of treatment, Δ*K*_max_ was 3.04 D (SD ± 0.75) in group 1, 2.38 D (SD ± 0.51) in group 2, 1.57 D (SD ± 0.35) in group 3, and 0.31 D (SD ± 0.20) in group 4. It is obvious that the change of *K*_max_ became smaller as the TCT increases. We found significant flattening of 3.04 D induced by CXL in the advanced subgroup (<400 *μ*m), which was more than in the mild to moderate subgroup. These results are similar to another study using accelerated CXL. It also showed a more striking effect on *K*_max_ reduction in thin versus thick corneas 6 months after CXL [14]. This phenomenon may be due to the following reasons: first, the main reason for the more striking flattening effect of CXL in thin corneas is the anterior localization of the CXL effect resulting in an overall higher relative proportion of crosslinked stroma in thin corneas. Second, there was a difference in the thickness of corneal stroma in mild to moderate patients versus advanced cases of keratoconus. Stroma is the major structural part of the cornea which maintains the bulk of the mechanical strength of the cornea while maintaining the high degree of transparency required for the light transmission. It consists of collagen types I, III, V, and VI that are synthesized by the keratocytes along with the proteoglycan macromolecules [15]. HRT II confocal microscopy showed clear vertical and horizontal transition zones with a slight spread of edema and a regular number of cells in the untreated corneal periphery and at depths 345 mm (range, 275–345 *μ*m). The deep corneal stroma beyond 350 *μ*m did not show any changes in endothelial density or morphology [16]. A three-year prospective nonrandomized open trial by in vivo corneal micromorphological microscopy found that there existed demarcation lines between treated and untreated stroma to a depth of 340 mm including epithelium [17]. Third, from the biomechanical view, it may be due to the localization of CXL effect in the anterior portion of the stroma [18]. Wollensak et al. found the collagen diameter in the treated rabbit corneas increased by 12.2% anteriorly and by 4.6% posteriorly, and the collagen diameter was significantly larger in the anterior stroma than in the posterior stroma [19]. Finally, thin cornea group was administered by hypoosmolar riboflavin solution until adequate thickness was achieved. The deepithelialized cornea can swell to double its normal thickness and creating collagen-free “lakes” [20]. Perhaps this allows ultraviolet light to become more convenient to permeate.

We found that there was a negative linear correlation between TCT and Δ*K*_max_. This has very important guiding significance before surgery, and it can predict the CXL effect.

With regard to TCT, there was a statistically thinning between preoperative and 1-year postoperative in both groups. Similar results were reported by Poli et al. in their 3-year prospective study of CXL [21]. In addition, a systematic review and meta-analysis which included 1171 participants with 1557 affected eyes found a slight decrease in CCT from baseline to 12 months post-CXL and then recovered to baseline after more than 18 months [22]. Perhaps the reason is the follow-up period was too short to be observed.

In addition, we found that ECD and IOP changed during the 12 months post-CXL (*P* < 0.05), but there were no changes in the size and shape of the endothelial cells, and the decrease in the number of the endothelial cells did not induce any endothelial cell-related complications such as corneal edema. There was no relationship between ECD changes and the change in TCT from preoperative to 6 months and 1 year postoperative, suggesting no clinical diminution in endothelial cell function from the procedure. The most common complication was corneal haze, which is temporary and does not disrupt vision. The postoperative temporary haze was often paracentral and compatible with good visual results. It may not be actually related to CXL itself but rather to the ongoing disease process and corneal remodeling [23].

In conclusion, this study has demonstrated the efficacy and safety of CXL with UVA-riboflavin in the stabilization of keratoconus. Keratoconic corneas with the thinnest pachymetry values less than 400 *μ*m had the greatest changes of maximum keratometry values. Advanced cases of progressive keratoconus seemed to obtain more benefits from the flatting effects of corneal CXL. There was a negative linear correlation between TCT and Δ*K*_max_ at the same time. However, further long-term follow-up studies with a larger number of participants are warranted.

## Figures and Tables

**Table 1:** Baseline, 6, and 12 months outcomes after CXL in all eyes (*n* = 123).

Parameter	Preop	6 mo postop	12 mo postop
TCT (*μ*m)	445.5 ± 48.7	421.6 ± 48.2^∗^	422.3 ± 45.9^∗^
*K*_max_ (D)	58.99 ± 9.99	57.24 ± 9.21^∗^	57.05 ± 9.23^∗^
*K*1 (D)	47.14 ± 4.45	46.83 ± 4.37^∗^	46.56 ± 4.25^∗^
*K*2 (D)	50.33 ± 5.73	49.84 ± 5.42^∗^	49.56 ± 5.34^∗^
UCVA (logMAR)	0.80 ± 0.36	0.72 ± 0.36^∗^	0.68 ± 0.35^∗^
BCVA (logMAR)	0.35 ± 0.33	0.25 ± 0.28^∗^	0.20 ± 0.26^∗^
IOP (mmHg)	9.97 ± 1.90	11.16 ± 2.92^∗^	11.34 ± 2.09^∗^
ECD (cells/mm^3^)	3048.32 ± 335.52	2982.70 ± 362.29^∗^	2951.48 ± 363.15^∗^

^∗^ *P* < 0.05, the difference was statistically significant.

**Table 2:** One-year outcomes of CXL by group.

Parameter	Group 1 (*n* = 31)		Group 2 (*n* = 41)		Group 3 (*n* = 36)		Group 4 (*n* = 15)	
	Preop	12 mo postop	Preop	12 mo postop	Preop	12 mo postop	Preop	12 mo postop
TCT (*μ*m)	376.7 ± 15.9	357.7 ± 15.8^∗^	429.8 ± 10.7	406.6 ± 18.8^∗^	470.8 ± 14.5	446.9 ± 16.5^∗^	515.3 ± 9.6	483.5 ± 5.1^∗^
*K*1 (D)	51.57 ± 6.09	50.71 ± 5.68	47.50 ± 3.25	46.87 ± 3.29^∗^	45.17 ± 2.38	44.76 ± 2.39	44.28 ± 1.24	43.83 ± 1.30^∗^
*K*2 (D)	55.74 ± 7.33	54.31 ± 6.67	51.15 ± 4.46	50.44 ± 4.47^∗^	47.55 ± 3.46	47.09 ± 3.37	46.62 ± 2.44	46.18 ± 2.74^∗^
*K*_max_ (D)	62.83 ± 9.24	59.20 ± 10.70^∗^	59.45 ± 6.90	57.18 ± 5.37^∗^	57.80 ± 8.58	55.33 ± 9.60^∗^	50.50 ± 4.53	50.95 ± 5.19
UCVA (logMAR)	0.90 ± 0.38	0.69 ± 0.43^∗^	0.93 ± 0.42	0.81 ± 0.35^∗^	0.91 ± 0.29	0.74 ± 0.30^∗^	0.53 ± 0.47	0.28 ± 0.26^∗^
BCVA (logMAR)	0.46 ± 0.48	0.21 ± 0.32^∗^	0.49 ± 0.29	0.29 ± 0.34^∗^	0.32 ± 0.33	0.15 ± 0.18^∗^	0.10 ± 0.20	0.08 ± 0.15^∗^
IOP (mmHg)	9.99 ± 2.49	10.93 ± 2.39^∗^	9.75 ± 1.33	12.25 ± 1.61^∗^	10.13 ± 2.00	10.90 ± 2.18	12.43 ± 1.45	13.58 ± 3.89^∗^
ECD (cells/mm^3^)	3151.33 ± 279.07	2807.33 ± 674.75^∗^	3090.81 ± 493.20	2874.54 ± 537.35^∗^	3036.50 ± 416.94	2942.91 ± 392.95^∗^	2870.33 ± 110.42	2805.68 ± 116.97^∗^

^∗^ *P* < 0.05, the difference was statistically significant between preop and 12 mo postop.

**Table 3:** Comparison of Δ*K*_max_ between the four groups at 6-month follow-up.

	Group 1	Group 2	Group 3	Group 4
*∆K*_max_ (D)	2.80 ± 0.61	2.02 ± 0.47	1.62 ± 0.31	0.05 ± 0.23
*χ* ^2^	16.476			
*P* value	0.001^∗^			

^∗^ *P* < 0.05, the difference was statistically significant.

**Table 4:** Comparison of Δ*K*_max_ between the four groups at 1-year follow-up.

	Group 1	Group 2	Group 3	Group 4
Δ*K*_max_ (D)	3.04 ± 0.75	2.38 ± 0.51	1.57 ± 0.35	0.31 ± 0.20
*χ* ^2^	17.603			
*P* value	0.001^∗^			

^∗^ *P* < 0.05, the difference was statistically significant.
